# Thyroid Metastases from Renal Cell Carcinoma: Review of the Literature

**DOI:** 10.1100/tsw.2010.55

**Published:** 2010-04-01

**Authors:** Alessandro Sindoni, Massimo Rizzo, Giovanni Tuccari, Antonio Ieni, Valeria Barresi, Letterio Calbo, Eugenio Cucinotta, Francesco Trimarchi, Salvatore Benvenga

**Affiliations:** ^1^ University of Messina, Italy; ^2^ Department of Human Pathology, Section of Clinical Oncology, University of Messina, Italy; ^3^ Department of Human Pathology, University of Messina, Italy; ^4^ Department of Human Pathology, Section of Surgery, University of Messina, Italy; Department of Clinical and Experimental Medicine and Pharmacology, Section of Endocrinology, University of Messina, Italy; ^6^ 6Program of Clinical and Molecular Endocrinology, A.O.U. Policlinico “G. Martino”, Messina, Italy

**Keywords:** thyroid nodules, kidney, renal cell carcinoma, metastasis

## Abstract

The thyroid gland is a rare site of clinically detectable tumor metastasis and kidneys are frequently the site of the parent malignancy. In the present review on thyroid metastases from renal cell carcinoma, cases were searched on PubMed by entering the strings: “renal carcinoma [or“hypernephroma”] AND thyroid metastasis/metastases”. Thus, we retrieved a total of 111 cases that were published between 1964 and 2007, a total that became 113 by adding two patients observed by us. The female to male ratio was 1.35:1. The primary renal cancer was almost always unilateral (90%) (with no significant side preference) and only rarely bilateral (9% in men, 4% in women), whereas bilaterality of thyroid metastases was relatively more frequent (28%). Thyroid metastasis from renal cancer was commonly single with a unique node that appears solid and hypoechoic at ultrasonography, approximately 9 years after nephrectomy. Concordance of lateralization was insignificantly greater for the right kidney/right thyroid lobe pattern (54%) than for the left kidney/left thyroid lobe pattern (40%), regardless of gender. Finally, survival was longer for women. Thyroid metastases, even if rare in the clinical practice, must be considered in the differential diagnosis of a thyroid nodule, particularly in patients who have a history of malignancies.

## INTRODUCTION

The appearance of a thyroid nodule in a patient with a primary, nonthyroid malignancy represents a diagnostic problem in that the nodule can be benign or malignant, and in the latter case, it may be a primary or metastatic cancer. Although the thyroid is richly supplied with blood, it is a rare site of metastasis. Indeed, metastases account for approximately 2% of thyroid malignancies; thyroid metastases are found in 0–13% of patients who have undergone thyroidectomy and in 0–13% of patients submitted to fine needle aspiration (FNA) biopsy. Authoptic studies demonstrated a higher prevalence: intrathyroidal metastases may occur in up to 24% of patients with widespread malignant lesions[1,2,3].

As summarized by Nakhjavani et al.[4] in nine autopsy series covering the years 1931–1969 with a number of cases ranging from 62 to 16,294, the rate of thyroid involvement ranged from 1.2% (total n = 16,294) to 24.2% (total n = 62). From an autopsy-based review on 1980 patients with metastatic neoplasms, intrathyroid metastases were detected in 8.6% of patients[1]. The primary malignancies causing the most frequent thyroid metastases were melanoma (39%), breast carcinoma (21%), and renal cell carcinoma (RCC) (12%). In contrast, the review by Haugen et al.[5] on an autopsy series of 253 patients found that breast (26%) and lung (25%) cancers were the most common primary malignancies causing thyroid metastases, while melanoma, RCC, and gastrointestinal tumors were found less frequently (11, 10, and 8%, respectively).

The largest clinical study from a single institution comes from the Mayo Clinic and covers the 10-year period between 1985 and 1994[4]. Of 43 patients with metastasis to the thyroid gland, the origin of the primary malignancy was identified in 41. The most frequent primary site was the kidney (14/41 or 34%), followed by lung and breast (7/41 or 17% each). The largest study on metastases of RCC to the thyroid gland is very recent[6]. This study includes 45 patients who underwent surgery for thyroid metastasis at 15 institutions of Germany and Austria between 1980 and 2007. This study is largely focused on analyzing the determinants of outcome.

Prompted by the observation of two patients at the Section of Endocrinology of our Hospital (to be described elsewhere for certain characteristics), we wished to review the literature on metastases of RCC to the thyroid and look for features that were not addressed in previous articles.

## METHODS

Our purpose was to highlight special features of both renal cancers and thyroid metastases in relation to gender and, particularly, ultrasonography characteristics. Cases were searched on PubMed by entering the strings: “renal carcinoma AND thyroid metastasis” or “renal carcinoma AND thyroid metastases” or “hypernephroma AND thyroid metastasis (or metastases)”. Thus, we retrieved a total of 111 cases that were published in the years 1964 to 2007[7–63], a total that became 113 by adding two patients that we had observed (Table 1). Except for three patients, the primary malignancy was clear cell carcinoma (Table 1).

Of the 113 patients, gender was specified for 94. Of these, 54 (57%) were women and 40 (43%) men, resulting in a F:M ratio of 1.35:1. Age was reported for 87 patients, and both age and gender for 83 (47 women and 36 men). Localization of the renal tumor was specified for 73 of these 83 patients (64.6% of the total of 113), plus another 29 patients with unknown age and/or gender. Localization of thyroid metastases was described in 71 cases with known age, sex, and localization of renal cancer, while for the other 25 patients, at least one of these three indices was missing.

## RESULTS

### Renal Tumor Side

Renal localization was predominantly unilateral, with no preference between the right or the left side (44.1 and 46.1%, respectively), being bilateral in only 9.8% of patients. The slightly greater preference for the right kidney in women compared to men (51 vs. 40%) was statistically insignificant ($p = 0.495$).

**TABLE 1**
**Summary of Case Reports of Kidney Cancers with Thyroid Metastases[a]**

| Case No. | Renal Localization | Thyroid Lobe Localization | | Gender | Age | Study Year | Ref. |
|---|---|---|---|---|---|---|---|
| 1 | Right | Right | CONCORDANT | M | 81 | 2004 | [11] |
| 2 | Right | Right | | M | 62 | 2006 | [30] |
| 3 | Right | Right | | M | 48 | 2007 | [40] |
| 4 | Right | Right | | M | 54 | 1996 | [46] |
| 5 | Right | Right | | M | 63 | 1999 | [50] |
| 6 | Right | Right | | M | 79 | 1999 | [50] |
| 7 | Right | Right | | M | n/s[b] | 2003 | [44] |
| 8 | Right | Right | | F | 62 | 2004 | [11] |
| 9 | Right | Right | | F | 47 | 1998 | [13] |
| 10 | Right | Right | | F | 50 | 2006 | [18] |
| 11 | Right | Right | | F | 56 | 2006 | [18] |
| 12 | Right | Right | | F | 71 | 2006 | [18] |
| 13 | Right | Right | | F | 62 | 1987 | [21] |
| 14 | Right | Right | | F | 87 | 2004 | [35] |
| 15 | Right | Right | | F | 58 | 1999 | [50] |
| 16 | Right | Right | | F | 52 | 1999 | [51] |
| 17 | Right | Right | | F | 54 | 1988 | [53] |
| 18 | Right | Right | | n/s[b] | 65 | 2000 | [14] |
| 19 | Right | Right | | n/s[b] | n/s[b] | 1999 | [16] |
| 20 | Right | Right | | n/s[b] | n/s[b] | 1981 | [17] |
| 21 | Right | Left | DISCORDANT | M | 61 | 2004 | [11] |
| 22 | Right | Left | | M | 32 | 2007 | [12][c] |
| 23 | Right | Left | | M | 68 | 2006 | [18] |
| 24 | Right | Left | | M | 77 | 2006 | [18] |
| 25 | Right | Left | | M | 62 | 2000 | [52] |
| 26 | Right | Left | | M | n/s[b] | 2003 | [44] |
| 27 | Right | Left | | F | 80 | 2004 | [11] |
| 28 | Right | Left | | F | 69 | 2006 | [18] |
| 29 | Right | Left | | F | 58 | 1975 | [43] |
| 30 | Right | Bilateral | | F | 35 | 1964 | [62] |
| 31 | Right | Bilateral | | F | 59 | 1964 | [62] |
| 32 | Right | Bilateral | | F | 45 | 2003 | [59] |
| 33 | Right | Bilateral | | F | 65 | 1964 | [62] |
| 34 | Right | Bilateral | | F | 57 | 1976 | [36] |
| 35 | Right | Bilateral | | F | 68 | 2001 | [57][c] |
| 36 | Right | Bilateral | | F | n/s[b] | 2003 | [44] |
| 37 | Right | Bilateral | | F | n/s[b] | 2003 | [44] |
| 38 | Right | Isthmus | | F | n/s[b] | 2003 | [44] |
| 39 | Right | Isthmus | | F | n/s[b] | 2003 | [44] |
| 40 | Right | n/s[b] | UNKN | M | n/s[b] | 1993 | [45] |
| 41 | Right | n/s[b] | | F | 79 | 2000 | [14] |
| 42 | Right | n/s[b] | | F | 63 | 1988 | [38] |
| 43 | Right | n/s[b] | | n/s[b] | 65 | 2000 | [14] |
| 44 | Right | n/s[b] | | n/s[b] | n/s[b] | 1999 | [16] |
| 45 | Right | n/s[b] | | n/s[b] | n/s[b] | 1989 | [42] |

*Table 1 continues*

| Case No. | Renal Localization | Thyroid Lobe Localization | | Gender | Age | Study Year | Ref. |
|---|---|---|---|---|---|---|---|
| 46 | Left | Left | CONCORDANT | M | 64 | 2007 | [37] |
| 47 | Left | Left | | M | 71 | 1995 | [28] |
| 48 | Left | Left | | M | 65 | 2006 | [49] |
| 49 | Left | Left | | M | 54 | 2000 | [52] |
| 50 | Left | Left | | M | 70 | 1964 | [62] |
| 51 | Left | Left | | M | 67 | 2008 | This study |
| 52 | Left | Left | | F | 55 | 2004 | [11] |
| 53 | Left | Left | | F | 50 | 2006 | [18] |
| 54 | Left | Left | | F | 69 | 2006 | [18] |
| 55 | Left | Left | | F | 74 | 2003 | [25] |
| 56 | Left | Left | | F | 48 | 2004 | [39] |
| 57 | Left | Left | | F | 37 | 1983 | [41] |
| 58 | Left | Left | | F | 72 | 1994 | [48] |
| 59 | Left | Left | | F | 77 | 2001 | [58] |
| 60 | Left | Left | | F | 60 | 2002 | [61] |
| 61 | Left | Left | | F | n/s[b] | 2003 | [44] |
| 62 | Left | Left | | n/s[b] | n/s[b] | 1988 | [27] |
| 63 | Left | Right | DISCORDANT | M | 70 | 2006 | [33] |
| 64 | Left | Right | | M | 77 | 2005 | [60] |
| 65 | Left | Right | | M | 66 | 1964 | [62] |
| 66 | Left | Right | | M | 53 | 1964 | [62] |
| 67 | Left | Right | | M | n/s[b] | 2003 | [44] |
| 68 | Left | Right | | F | 65 | 2007 | [7] |
| 69 | Left | Right | | F | 68 | 2006 | [18] |
| 70 | Left | Right | | F | 72 | 2006 | [18] |
| 71 | Left | Right | | F | 66 | 2002 | [19] |
| 72 | Left | Right | | F | 59 | 2000 | [52] |
| 73 | Left | Right | | F | 53 | 2004 | [54] |
| 74 | Left | Right | | F | n/s[b] | 2003 | [44] |
| 75 | Left | Right | | n/s[b] | n/s[b] | 1999 | [16] |
| 76 | Left | Bilateral | | M | 78 | 1999 | [9] |
| 77 | Left | Bilateral | | M | 66 | 2004 | [11] |
| 78 | Left | Bilateral | | M | 54 | 2006 | [15] |
| 79 | Left | Bilateral | | M | 54 | 2006 | [18] |
| 80 | Left | Bilateral | | M | 56 | 2004 | [47] |
| 81 | Left | Bilateral | | M | 52 | 2003 | [56] |
| 82 | Left | Bilateral | | M | 79 | 2009 | This study |
| 83 | Left | Bilateral | | F | 76 | 2007 | [8] |
| 84 | Left | Bilateral | | F | 60 | 2004 | [11] |
| 85 | Left | Bilateral | | F | 59 | 2006 | [18] |
| 86 | Left | Bilateral | | F | 58 | 2006 | [33] |
| 87 | Left | Bilateral | | F | n/s[b] | 2003 | [44] |
| 88 | Left | n/s[b] | UNKN | n/s[b] | 58 | 2000 | [34] |
| 89 | Left | n/s[b] | | n/s[b] | n/s[b] | 1998 | [10] |
| 90 | Left | n/s[b] | | n/s[b] | n/s[b] | 1983 | [23] |
| 91 | Left | n/s[b] | | n/s[b] | n/s[b] | 1999 | [26][c] |
| 92 | Left | n/s[b] | | n/s[b] | n/s[b] | 1991 | [29] |

*Table 1 continues*

| Case No. | Renal Localization | Thyroid Lobe Localization | | Gender | Age | Study Year | Ref. |
|---|---|---|---|---|---|---|---|
| 93 | Bilateral | Bilateral | | M | 62 | 2006 | [18] |
| 94 | Bilateral | Bilateral | C | M | 55 | 2006 | [22] |
| 95 | Bilateral | Bilateral | O | M | 71 | 1964 | [62] |
| 96 | Bilateral | Bilateral | N | n/s[b] | n/s[b] | 1999 | [16] |
| 97 | Bilateral | Bilateral | C | n/s[b] | n/s[b] | 2007 | [32] |
| 98 | Bilateral | Bilateral | | n/s[b] | n/s[b] | 2007 | [32] |
| 99 | Bilateral | Left | D | F | 60 | 1991 | [31] |
| 100 | Bilateral | Left | I | F | 61 | 2006 | [33] |
| 101 | Bilateral | Left | S | n/s[b] | n/s[b] | 1999 | [16] |
| 102 | Bilateral | n/s | | n/s[b] | n/s[b] | 1998 | [10] |
| 103 | n/s[b] | Right | | M | 76 | 1999 | [50] |
| 104 | n/s[b] | Right | | F | 81 | 1991 | [55] |
| 105 | n/s[b] | Left | U | F | 82 | 1995 | [28] |
| 106 | n/s[b] | Left | N | F | 67 | 1998 | [63] |
| 107 | n/s[b] | Left | K | n/s[b] | 61 | 2007 | [24] |
| 108 | n/s[b] | Bilateral | N | M | 50 | 1964 | [62] |
| 109 | n/s[b] | n/s[b] | O | M | 71 | 1982 | [20] |
| 110 | n/s[b] | n/s[b] | W | M | 58 | 1982 | [20] |
| 111 | n/s[b] | n/s[b] | N | M | 53 | 1982 | [20] |
| 112 | n/s[b] | n/s[b] | | F | 69 | 1982 | [20] |
| 113 | n/s[b] | n/s[b] | | F | 68 | 1982 | [20] |

[a] The studies by Nakhjavani et al.[4] and Iesalnieks et al.[6] do not appear in Table 1 because they provide very limited data. In the German-Austrian study[6], the pertinent data provided were that there were 45 patients (21 men and 24 women) of mean age 67. The 45 patients include five patients published previously[32,33], and appearing in Table 1. In the American study[4], the pertinent data provided were that there were 14 patients (11 men and three women) of mean age 68.

[b] Not stated in report.

[c] Renal cancer was clear cell carcinoma except in three patients: carcinoid[12], papillary[26], and chromophobe carcinoma[57].

## Age

Age did not differ between women (62.6 ± 11.4) and men (63.3 ± 10.8). The most frequent decade was the $7^{th}$ (61–70 years), followed by the $6^{th}$ (51–60), as they represented 34.1 and 30.7% of the 87 patients, respectively. However, women in the $6^{th}$ decade were slightly more prevalent than in the $7^{th}$ decade (35.4 and 27.1%), just the opposite than in men (25 and 36.1%). Overall, the distribution over the $4^{th}$ through the $9^{th}$ decades was not statistically significant in women compared to men (degrees of freedom = 5, $\chi^2$ = 0.928, $p$ = 1.0).

## Thyroid Ultrasonography (US)

Of the total of 113 patients, US characteristics of thyroid metastases were specified only for 32 (28.3%) and gender for 22/32; in 1/32[7], the authors did not specify whether the solid metastasis was hyperechoic or hypoechoic.

Most thyroid nodules were hypoechoic (18 or 58%) and only a few were hyperechoic (2 or 6.5%), while approximately one-third were mixed (11 or 35.5%) (Table 1). Taking into account the limited size of this sonographic series, intergender difference between these three types of nodules was statistically insignificant.

Concerning the size of the thyroid metastasis, at least the largest (or maximum) diameter was specified in 19 cases (nine men and 10 women) and it ranged from 11 to 60 mm (34.7 ± 12.3, mean ± SD). In women, the maximum diameter ranged from 25 to 60 mm (37.7 ± 11.4), whereas in men it ranged from 11 to 52 mm (31.4 ± 13.2, $p$ = 0.677 vs. women). Similarly, upon setting cut-off values for the largest diameter of thyroid metastases, no cut-off values distribution in men differed significantly from distribution in women. For instance, the proportions of thyroid metastases having the largest diameter <40 mm were 67% in men and 40% in women, while the proportions of thyroid metastases having the largest diameter ≥40 mm were 33 and 60%, respectively (degrees of freedom = 1; $\chi^2$ = 0.49, $p$ = 0.48). Because the largest diameter of the corresponding primary renal cancer was specified in only two cases[56,58], no correlation analysis between the size of the primary tumor and the thyroid metastasis was possible.

## Interval from Nephrectomy to the Diagnosis of Thyroid Metastasis

The time interval from nephrectomy to the diagnosis of metastatization to the thyroid gland was specified for 87 patients (30 men, 47 women, and 10 unknown gender). Thyroid metastases were synchronous in eight patients (9.2%) and metachronous in 79 (90.8%). There was no significant sex difference ($\chi^2$ = 0.086, $p$ = 0.769) between synchronous (four men, four women) and metachronous (26 men and 43 women) metastases. Similarly, there was no significant sex difference in the interval between nephrectomy and diagnosis of thyroid metastasis in men (8.7 ± 6.4 years [range: 1.0–19.5]) vs. women (8.8 ± 5.9 years [range: 0.25–26.3]). The proportions of thyroid metastases having an interval from nephrectomy of ≤10 years, 11–20 years, and >20 years in women vs. men were 62.8 vs. 52%, 32.6 vs. 48%, and 4.7 vs. 0%, respectively (degrees of freedom = 2; $\chi^2$ = 2.462, $p$ = 0.292).

## Thyroid Metastases Localization

When known (n = 101), metastatization to the thyroid consisted of a single nodule in 78 patients (77%) and multiple nodules in 23 (23%). When specified, (4/23 patients) the number of metastatic thyroid multiple nodules ranged from two to three in either lobe. Overall, thyroid metastases were localized to the right and left lobe with similar frequency (37 and 34%); the remaining 29% having thyroid metastases in both thyroid lobes or in the isthmus. When considering only the 78 solitary nodules, results did not change (right lobe = 39%, left lobe = 35%).

The thyroid metastases showed no significant intergender difference ($p$ = 0.94) concerning lateralization. The development of intrathyroid disease recurrence after surgical removal of thyroid metastasis/metastases was already treated by another study[6].

## Concordance of Laterality between the Renal and the Thyroid Lesions

Excluding two patients with a thyroid metastasis localized in the isthmus, in 88 patients it was possible to determine such concordance that was (1) a renal cancer localized in the right kidney causing one or more thyroid metastases in the right lobe, (2) a renal cancer of the left kidney metastasizing to the left thyroid lobe, (3) a renal cancer localized in the two kidneys metastasizing to both thyroid lobes. Of these 88 patients, 43 (48.9%) showed concordance and 45 (51.1%) showed discordance. Of the 43 concordant patients, 20 (22.7%) had a right/right localization, 17 (19.3%) a left/left localization, and 6 (6.8%) a bilateral/bilateral localization. Of the 45 patients with discordance, the most frequent patterns were left kidney/right thyroid lobe (13/45 or 28.9%) and left kidney/bilateral thyroid (12/45 or 26.7%), while the rarest was bilateral kidneys/right thyroid lobe (0%). Worthy of note, the two patients (No. 38 and No. 39)

with the isthmus localization of the thyroid metastasis had a right-sided renal cancer and were women (Table 1).

Excluding the said two patients with an isthmic localization of the thyroid metastasis, of the 37 patients with a right-sided renal cancer for which the localization of thyroid metastatization was specified (21 women, 13 men, and three unknown gender), 20 (54%) had a concordantly right-sided thyroid metastasis and 17 (46%) a discordantly left-sided or bilateral thyroid metastatization. The rate of the right/right concordance was similar in men and in women (7/13 or 53.8% vs. 10/21 or 47.6%, $p = 1.0$ by Fisher's exact test). Interestingly, 8/8 patients with the discordant right-sided renal cancer and bilateral thyroid metastases were women.

Of the 42 patients with a left-sided renal cancer for which the thyroid metastasis localization was specified (22 women, 18 men, and two unknown gender), 17 (40%) had a concordantly left-sided localization of the thyroid metastatization and 25 (60%) a discordantly right-sided or bilateral thyroid metastatization. The rate of the left/left concordance in women vs. men was 45.5% (10/22) vs. 33.3% (6/18) ($p = 0.53$ by Fisher's exact test). Thus, concordance was greater if the primary cancer was right sided rather than left sided both in men (53.8 vs. 33.3%) and women (47.6 vs. 45.4%).

Of the nine patients with a bilateral kidney cancer for which the thyroid metastatization was known (two women, three men, and four unknown gender), six (67%) had a concordant localization of metastases in the two thyroid lobes. The rate of the bilateral/bilateral concordance was different in women vs. men (0/2 vs. 3/3), but not significantly so ($p = 0.10$ by Fisher's exact test) due to the very small numbers.

Age did not differ between the patients with concordant thyroid metastatization and the patients with a discordant localization of metastases (Fig. 1). In the concordant group, men were consistently 4.3–5.0 years older than women, whereas in the discordant group, this gap dropped to 1.2 years (Fig. 1). However, the intergender comparison of age between the two groups yielded no statistical significance (Fig. 1).

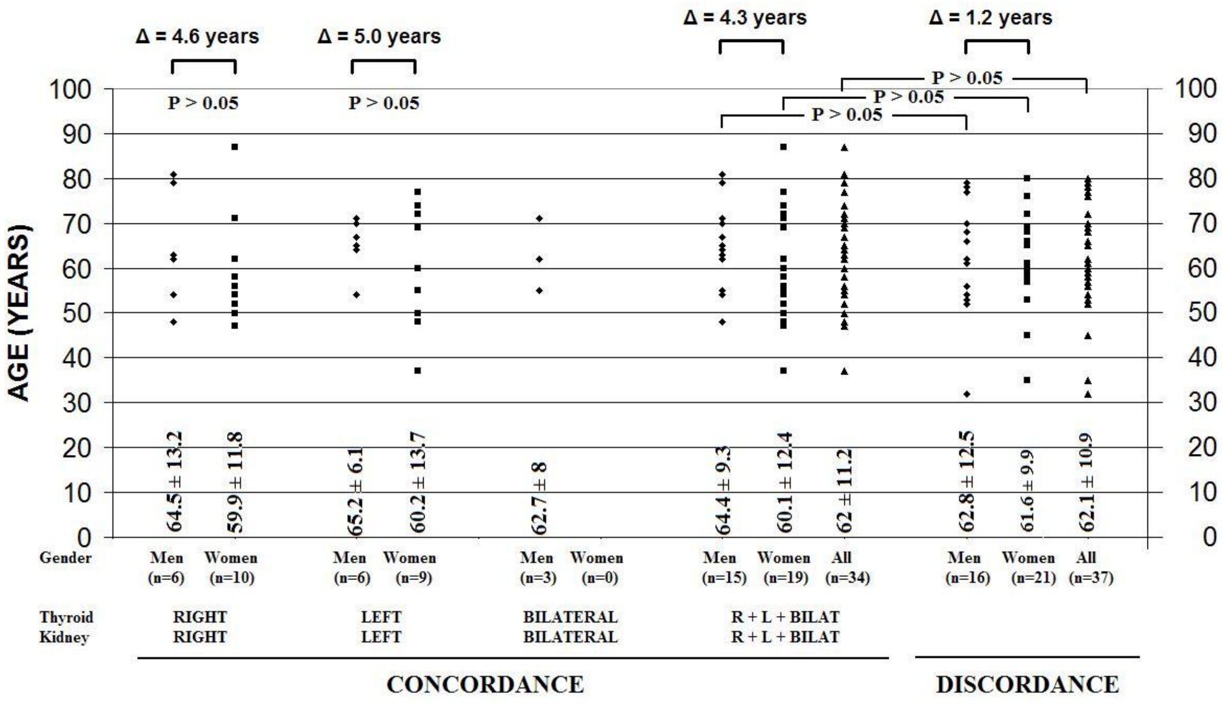

**FIGURE 1.** Age at presentation in patients stratified based on concordance of lateralization between the primary kidney cancer and the thyroid metastases.

## Survival Analysis

Follow-up data were specified for 61 cases (34 women and 27 men), all affected by RCC. Excluding one male patient who died because of dyspnea 1 day after thyroidectomy[62], there was no operative morbidity or mortality associated with the thyroidectomy. We used the Kaplan-Meier method to estimate the probability of survival from the time of the diagnosis of thyroid metastases from RCC. Median survival was 62 months, while overall survival rates for the whole study population (n = 60) at 2, 4, 6, 8, and 10 years were 70.1, 54.4, 39.9, 34.2, and 11.4%, respectively.

The survival rates in women vs. men were 77.2 vs. 61.3% (2 years), 67.2 vs. 39.7% (4 years), 67.2 vs. 34.1% (6 years), 50.4 vs. 22.7% (8 years), and 25.2 vs. 0% (10 years) ($p = 0.021$ by log-rank Mantel-Cox test) (Fig. 2). This gender difference was not significant in the subgroup of patients aged <70 years ($p = 0.183$), but it was trendwise significant in the subgroup aged ≥70 years ($p = 0.064$) (Fig. 2). In the first subgroup, the survival rates at 2, 4, 6, 8, and 10 years in women vs. men were 76.6 vs. 67%, 68.9 vs. 55.9%, 68.9 vs. 33.5%, 51.7 vs. 33.5%, and 25.8 vs. 0%, respectively. In the second subgroup, the survival rates at 2 and 4 years in women and men were, 75 vs. 34.5% and 75 vs. 17.3%, respectively.

## Nonthyroidal Metastases

In 31 patients (15 women, 15 men, and one unknown gender), the authors reported the presence of nonthyroidal metastases[7,8,9,11,12,13,15,22,23,28,30,31,33,45,47,49,51,52,56,62]. Secondary nonthyroidal lesions in women vs. men were 27.3 vs. 21.7% (lungs), 18.2 vs. 8.7% (pancreas), 13.6 vs. 26.1% (skeletal), 13.6 vs. 13% (lymph nodes), 9.1 vs. 8.7 (liver), and 9.1 vs. 4.3% (adrenal glands); the remaining metastases being localized in other organs or tissues. The twofold greater prevalence of pancreas metastases in women and the twofold greater prevalence of skeletal metastases in men were statistically insignificant (degrees of freedom = 6; $\chi^2 = 2.424$, $p = 0.658$).

## DISCUSSION

RCC is known for its tendency to cause metastases unpredictably. If clinically apparent, the thyroid metastasis is not different from the generality of thyroid nodules, in that it consists of a thyroid nodule that is scintigraphically "cold" (unable to trap the radioisotope) and, most frequently, ultrasonographically solid. At FNA cytology, it is characterized by foamy clear cells with nuclear atypia and cytoarchitectural disorder. Detection of thyroid metastasis before the primitive tumor is a rare event[4,64]. Metastases usually appear after the primary cancer (metachronous metastases), even more than 10 years after nephrectomy. For instance, in a series of 14 patients reported by Nakhjavani et al.[4], one patient developed metastases 26 years after diagnosis of renal cancer; at the other extreme, another patient developed thyroid metastases only 1 month later. Metachronous metastases appear in over 50% of patients who were treated with radical nephrectomy[65]. Possible factors suggesting the slow development of thyroid metastases are the filtery activity of lungs, the high concentration of oxygen and iodine in the thyroid, and the local cytoregulating effect of the thyroid hormones[66]. According to Boles and Cerny[67], it is the Batson's venous plexus between the vertebral and epidural venous system that facilitates this spread. These venous systems are valveless and offer an easy way for metastatic cells to spread with low resistance. Increase in intra-abdominal and thoracic pressure causes retrograde flow in these veins; cancerous kidney cells can bypass the pulmonary capillary filtration and metastasizes in the head and neck. This pathogenetic suggestion has been recently reproposed by Kancherla et al.[68]. RCC typically invades the draining renal vein, entering the inferior vena cava, passing through the heart and lung to enter the systemic arterial system, only to access the thyroid gland via the thyroid arteries. Based on this route, no difference is to be expected in the pattern of thyroid metastasis according to gender or the side of the primary kidney tumor.

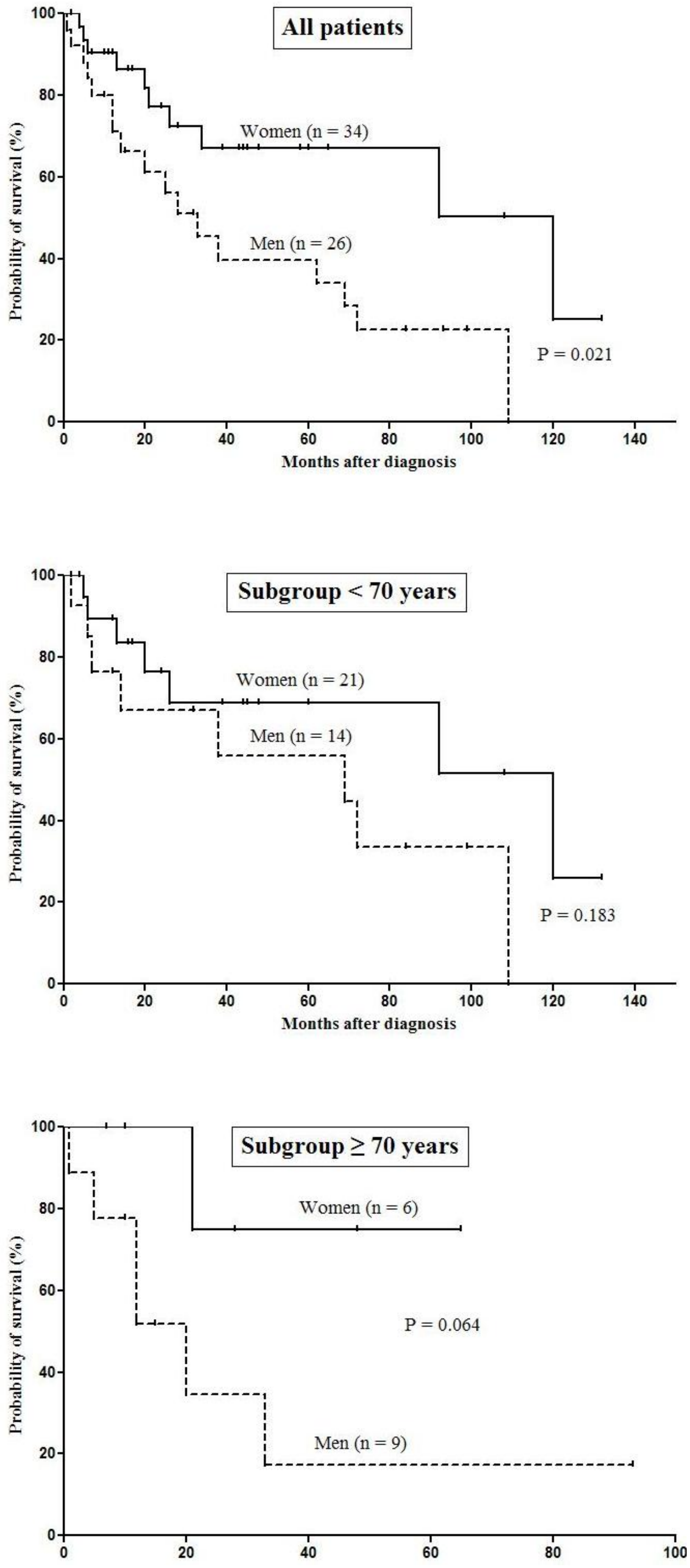

**FIGURE 2.** Influence of gender on survival of all patients and two subgroups based on age.

However, some phenomena for which we have no immediate explanation might be purely random. First, the left-sided RCC metastasizes insignificantly more frequently than the right-sided RCC to the two thyroid lobes (29 vs. 20%). Second, while in the bilaterally metastasizing left-sided RCC, the M:F ratio is 7:5 (1.40:1); in the bilaterally metastasizing right-sided RCC, the M:F ratio is 0:8. Third, while the female gender is associated with equal chances of side concordance (right thyroid lobe metastasis from the right kidney = 48%, left lobe metastasis from left kidney = 45%), the male gender is associated with greater chances of side concordance if the primary tumor is right sided (right lobe metastasis from the right kidney = 54%, left lobe metastasis from left kidney = 33%). This gender difference is amplified concerning concordance of the bilateral RCC, because bilateral RCC resulted in bilateral metastases only in men (3/3), but not in women (0/2), where localization of the metastasis was left thyroid lobe. Finally, the concordantly metastasizing RCC patients are a few years younger than the discordantly metastasizing RCC patients, the difference in age being accounted for by men.

Two prognostic factors for thyroid metastases in patients with RCC have been identified: (1) presence of an isolated metastasis and (2) treatment. Prolonged survival was reported in patients with solitary thyroid metastases[6]. Kierney et al.[69] reported 1-, 3-, and 5-year disease-free survivals of 77, 59, and 31%, respectively, for treated patients compared with a 36, 17, and 1.7% rate for patients with untreated metastases from a historical series.

In our study, we found no gender-related survival difference in the subgroup of patients aged <70 years ($p = 0.183$), but it was trendwise significant in the subgroup aged ≥70years ($p = 0.064$) (Fig. 2): this gender-specific difference was not present in the group <70 years where gender-specific biology does not play out as much as in the group ≥70 years (Fig. 2).

It is controversial whether a total thyroidectomy is obligatory in patients with metastatic RCC. No differences were observed between total and subtotal thyroidectomy in six large studies comprising 85 patients[4,17,18,44,70,71], approximately half of whom underwent total thyroidectomy.

Surgical treatment of solitary thyroid metastases is recommend because patients affected by a single thyroid metastasis have a good prognosis[70]. On the contrary, patients with disseminated disease have a poor prognosis, and surgical treatment of these patients is indicated as a life-saving measure or for the palliation of significant compressive symptoms[70]. In patients who cannot be thyroidectomized, methods to prolong survival include medical therapy, immunotherapy (e.g., interferon-α), multikinase inhibitors (sunitinib, sorafenib, axitinib, pazopanib), antivascular endothelial growth factor agents (bevacizumab), and mammalian target of rapamycin inhibitors (temsirolimus, everolimus).

Survival is short (mean of 8 months) for patients with synchronous thyroid metastases, but it is quite longer (mean of 10 years) for patients with metachronous thyroid metastases. Indeed, the longer the interval of time between the diagnosis of RCC and the appearance of thyroid metastasis, the better the prognosis[44].

The histopathologic distinction between a clear cell thyroid tumor and a renal cancer metastasis in the thyroid may be quite difficult, since the presence of follicles containing blood rather than colloid is not so common as indicated by Carcangiu et al.[72].

While histochemical stain for glycogen may be misleading because both kidney tumors and thyroid primitive lesions may be reactive, some help can be obtained by immunohistochemistry, particularly immunoreactivity for thyroglobulin (Tg). Indeed, a positive immunoreaction for Tg suggests the clear cell primary thyroid tumor, while a negative immunoreaction should indicate a metastasis from RCC. Lack of immunoreactivity for calcitonin and CEA together with a detailed clinical history are the most important clues for the correct diagnosis of a metastatic tumor[63]. In one of our cases (No. 82 in Table 1), immunohistochemical investigation was very helpful since the immunoreactive positive pattern regarding EMA, low molecular weight CK, and vimentin were consistent with a renal origin of neoplastic nodules.

## CONCLUSION

RCC is the most frequent malignancy of the kidney. Upon reviewing the literature, we found that kidney cancer has a slight preference for the right kidney among women and the left kidney among men. Thus, the left kidney localization of RCC in our two male patients agrees with the literature.

The intrathyroid metastases from renal cancer appear most frequently as hypoechoic solid nodules at US. In half of the cases, there is a concordance of laterality between RCC and thyroid metastasis.

Genders may differ for outlook, because survival in women was significantly longer than men.

Thyroid metastases, even if rare in clinical practice, must be considered in the differential diagnosis of a thyroid nodule, particularly in patients who have a history of nonthyroid primary malignancies. In the particular case of renal cancer, the primary kidney tumor could have been diagnosed and surgically treated even over 2 decades earlier than the appearance of the thyroid metastasis/metastases as single/multiple thyroid nodule/nodules.

Surgical treatment of solitary thyroid metastases is recommended because bilateral metastases recur in over one-fourth of patients, and prognosis is good as long as the thyroid metastasis is a single nodule. In contrast, patients with disseminated disease have a poor prognosis and their indication for thyroidectomy is palliative care of compressive symptoms. In patients surgically untreatable, the prolonged survival can be achieved by adjuvant medical therapy.
